# Influence of processing steps on the fate of ochratoxin A, patulin, and alternariol during production of cloudy and clear apple juices

**DOI:** 10.1007/s12550-021-00443-x

**Published:** 2021-10-25

**Authors:** Husam Ibrahem Aroud, Bianca May, Helmut Dietrich, Ralf Schweiggert, Sabine Kemmlein

**Affiliations:** 1 Berlin, Germany; 2 Institute of Beverage Research, Analysis and Technology of Plant-Based Foods, Geisenheim University, Von-Lade-Str. 1, 65366 Geisenheim, Germany; 3 Department of Safety in the Food Chain, BfR - Federal Institute for Risk Assessment, Max-Dohrn-Str. 8-10, 10589 Berlin, Germany; 4 Department Method Standardisation, Reference Laboratories, Resistance To Antibiotics, BVL - Federal Office of Consumer Protection and Food Safety, Diedersdorfer Weg 1, 12277 Berlin, Germany

**Keywords:** Patulin (PAT), Alternariol (AOH), Ochratoxin A (OTA), Apple juice, Processing chain, Fining agent

## Abstract

Mycotoxins are frequently found in fruits and fruit juices. However, data about occurrence and fate of mycotoxins along the fruit juice processing chain are currently insufficient. Herein, a liquid chromatographic/tandem mass spectrometric (LC–MS/MS) multi-mycotoxin method was developed and applied to investigate the effect of technological unit operations on the fate of three of the most relevant mycotoxins along the processing chain for cloudy and clear apple juice, namely patulin (PAT), ochratoxin A (OTA), and alternariol (AOH). Raw juice obtained directly after dejuicing was spiked with the aforementioned mycotoxins at pilot-plant scale prior to subjecting it to different technological unit operations. Regarding clear apple juice production treatment with a pectinolytic enzyme preparation, and pasteurization were insignificant for mycotoxin reduction, but fining with subsequent filtration was effective, although the mycotoxins showed different affinity towards the tested agents. The most effective fining agent was activated charcoal/bentonite in combination with ultrafiltration, which removed OTA (54 µg/L) and AOH (79 µg/L) to not quantifiable amounts (limit of quantification (LOQ) 1.4 and 4.6 µg/L, respectively), while PAT was reduced only by 20% (from 396 to 318 µg/L). Regarding cloudy apple juice production, all studied processing steps such as centrifugation and pasteurization were ineffective in reducing mycotoxin levels. In brief, none of the common steps of clear and cloudy apple juice production represented a fully effective safety step for minimizing or even eliminating common mycotoxins. Thus, ensuring the sole use of sound apples should be of utmost importance for processors, particularly for those manufacturing cloudy juices.

## Introduction

The occurrence of mycotoxins in fruit juice is mostly attributed to the use of fruits which are already contaminated with mycotoxins. Fungal invasion of fruits can happen directly in the field or during the postharvest storage of the fruits. Beside aflatoxins, the mycotoxins patulin (PAT), ochratoxin A (OTA), and *Alternaria* toxins are the most common mycotoxins found in fruits and derived products. In the case of apple products, especially PAT is considered to be a major concern for food safety and many studies have been carried out to screen the presence of this mycotoxin (e.g. Oteizaa et al. 2017; Iha and Sabino 2008; Poapolathep et al. 2017; Hammami et al. 2017; Zouaoui et al. 2015; Rice et al. 1977). For instance, 66% of the analysed apple juices from the Spanish market and 35% of juices from the Italian market were tested positive for PAT with levels ranging from 0.7 to 118.7 µg/L (mean 19.4 µg/L) and 1.6 to 55.4 µg/kg (mean 9.3 µg/kg), respectively (Murillo-Arbizu et al. 2009; Spadaro et al. 2007). In an Argentinian study, PAT was reported to occur at levels above the limit of detection (LOD) in 50% of the analysed apple purees at an average concentration of 123 µg/kg (Funes and Resnik 2009). However, other fruits like cherries, strawberries, and raspberries are described to contain PAT, too (Drusch and Ragab 2003). PAT is produced by *Aspergillus*, *Penicillium*, and *Byssochlamys* (Steiman et al. 1989) and is assumed to be genotoxic and to cause immune suppressive effects (e.g. Schumacher et al. 2005; Zhou et al. 2009; Puel et al. 2010; Al-Hazmi 2014). The International Agency for Research on Cancer (IARC) has classified patulin as category 3, not classifiable regarding its carcinogenicity to humans (IARC 1987). Hence, maximum levels have been established by the European Commission (EC) for this mycotoxin in apple juice (50 µg/kg); apple products, e.g. puree (25 µg/kg); and infant food (10 µg/kg) (EC 2006a).

The mycotoxin OTA is produced by *Aspergillus* and *Penicillium* (Lai et al. 1970; Varga et al. 1996; Larsen et al. 2001). It is genotoxic and is classified as a possible human carcinogen (group 2B) by the IARC (1993). Many studies suggested that OTA is the cause of Balkan endemic nephropathy (BEN) (Krogh et al. 1977; Radić et al. 1997). This mycotoxin is mainly found in grape-based products, e.g. grape juice, raisins, vinegar, and red or white wines. For instance, OTA was found in 29% of red wines and 23% of white wines from the Canadian market, but also in 6.5% of red grape juices and in 4% of white grape juices (Ng et al. 2004). Apples, strawberries, and cherries can also contain this mycotoxin (0.41 µg/kg, 1.44 µg/kg, 2.71 µg/kg, respectively) (Engelhardt et al. 1999).

*Alternaria* toxins such as alternariol (AOH), alternariol monomethyl ether (AME), altenuene, and tenuazonic acid (TeA) are produced by many species of the genus *Alternaria* being well known to infest apples (Harteveld et al. 2014). *Alternaria alternata* is considered as the predominant cause of mouldy core in apple. It is known to recover even after fungicide treatment of the fruits and to produce the genotoxic AOH (Pahlke et al. 2016; Reuveni et al. 2002; EFSA 2011). AOH and AME are the major mycotoxins in infected apples, while TeA and AME are mainly found in oranges and lemons (Stinson et al. 1981). A study of Spanish apple juice concentrates showed detectable amounts of AOH (1.4–5.4 µg/L) and AME (1.7 µg/L) in 50% of the analysed samples (Delgado and Gomez-Cordoves 1998). Another study confirmed the occurrence of AOH and AME in red and white grape juice and in cranberry juice, although at low concentrations (Scott et al. 2006).

Due to the toxicity and the wide occurrence of the aforementioned mycotoxins, studies on how to minimize their frequency or completely avoid their occurrence are of utmost interest. Mycotoxin reduction might already start with the selection of apple varieties having low vulnerability regarding PAT-producing strains (Watanabe and Ayugase 2009; Cunha et al. 2014). Good management practices in apple orchards, a preharvest-application of plant elicitors to induce defence mechanisms, or the selection of resistant apple varieties are other, possible mitigation strategies as reviewed by Zhong et al. (2018). Furthermore, pre-processing treatments like sorting and washing the fruit as well as trimming the mouldy parts are well known to effectively reduce the content of PAT (Sydenham et al. 1995). The additional use of chemical additives in the washing solution, like chlorine, chlorine dioxide, hydrogen dioxide, or ozonated water, can enhance the effectivity by not only reducing PAT itself, but also mycotoxin producing fungi (Ioi et al. 2017).

Also, the subsequent processing steps during apple juice production are described to have a further impact on mycotoxin levels. Generally, apple juice is produced through a multiple-stage process which includes several mechanical, and physical treatments, as well as the use of enzymes and, if necessary, fining agents. The processing is aligned to the desired product and the manufacturer’s equipment. While centrifugation and pasteurization of the freshly pressed juice are sufficient to produce a cloudy juice, further steps have to be conducted to obtain a clear and stable product. In this context, stability means a long-term clear product without visually noticeable polyphenolic, proteinaceous, pectic, and other hazes. In order to remove the respective colloids and compounds prone to precipitation, a treatment with pectinolytic enzyme preparations for partially degrading pectic colloids is carried out prior to the subsequent so-called fining, i.e. the adsorptive clarification using fining agents. Common fining agents include bentonite with either activated charcoal or colloidal silica and gelatine as well as the recently in the juice industry established plant proteins such as pea and potato proteins (Könitz 2014; Zobus et al. 2016). Subsequently, a filtration step, e.g. precoat filtration, sheet filtration, or ultrafiltration, yields the desired clarified product. Earlier studies, mainly focusing on PAT, demonstrated mycotoxin-reducing effects during these processing steps. However, the effects described are partly contradictory, e.g. the influence of pectinase treatment and filtration (Welke et al. 2009; Bissessur et al. 2001), or the stability of PAT against heat treatment (Kadakal and Nas 2003; Welke et al. 2009). The additional use of ascorbic acid (AA) can also effectively reduce PAT. Studies, analysing the effect of AA on PAT in cloudy apple juice, showed a 60% reduction of PAT (when using 0.25 or 4% AA) on a laboratory scale and a 50% reduction (when using 0.1% AA) on a technical scale (El Hajj Assaf et al. 2019, 2020). However, both studies had different outcomes on the influence of the presence of oxygen on the effectiveness of AA in reducing PAT.

Since results about the influence of processing steps are controversial, further studies are required to understand the effect of juice processing on PAT. Furthermore, there is still a lack of information on the impact of processing on the other mycotoxins as well as the influence of new processing aids, like plant proteins.

**Fig. 1:**
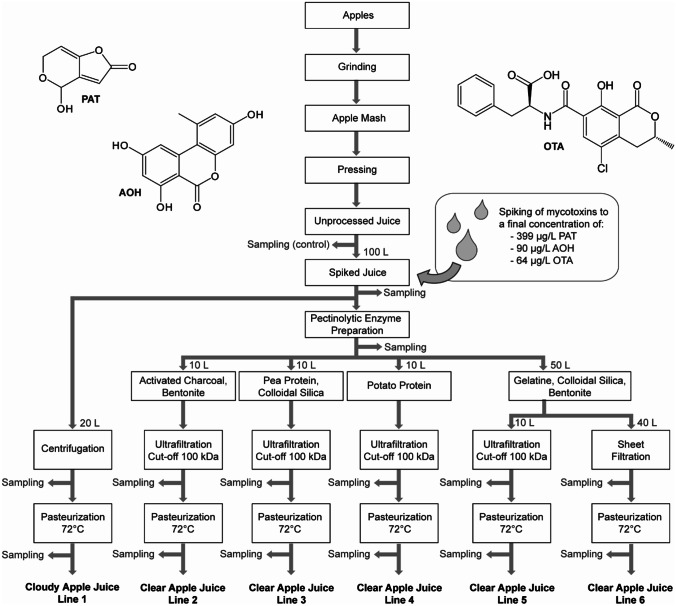
General processing scheme for the production of cloudy and clear apple juices following six different lines at pilot-plant scale. The point of mycotoxin addition, the sampling points, and the specific volumes used for the experiments are indicated

In this study, a newly developed liquid chromatographic/tandem mass spectrometric (LC–MS/MS) multi-mycotoxin method was applied to reliably and rapidly determine the concentrations of the most relevant mycotoxins, i.e. PAT, OTA, and AOH, in clear and cloudy apple juices and all intermediate products occurring along the production line (Fig. 1). Therefore, the effects of the common processing steps on the mycotoxin levels were examined, including enzymatic treatment (pectinolytic enzyme preparation), centrifugation, fining (with gelatin/colloidal silica/bentonite; bentonite/activated carbon; pea or potato protein), filtration (sheet filtration and ultrafiltration), and pasteurization.

## Materials and methods

### Materials, chemicals and reagents

Apples of mixed cultivars for laboratory juice processing were obtained from the Institute of Pomology (Geisenheim University), while apples for the larger pilot-plant scale were purchased from a local fruit and vegetable wholesaler (VOG, Ingelheim, Germany). For juice clarification, a pectinolytic enzyme preparation (Fructozym P), bentonite (Bento UF), colloidal silica (Klarsol Speedfloc; Klarsol 30), and gelatin (ErbiGel, 90–100 Bloom) were obtained from Erbslöh (Geisenheim, Germany), while activated charcoal (SIHA Aktivkohle UF), pea protein (SIHA Pea Protein), and potato protein (SIHA Potato Protein) were from Eaton (Dublin, Ireland).

Liquid chromatography (LC) solvent methanol (MS), ammonium acetate, and glacial acetic acid (p.a.) were purchased from Sigma–Aldrich (Darmstadt, Germany). LiChrosolv LC/MS hyper grade quality methanol and acetonitrile, ammonium acetate, and acetic acid (100%) were purchased from Merck (Darmstadt, Germany) for the preparation of the standards. For all experiments, analytical grade water (0.055 μS/cm) was generated from a Milli-Q system (Merck, Darmstadt, Germany). Mycotoxins, which were used in the spiking experiments, were purchased from Santa Cruz Biotechnology (Dallas, USA). Certified standards of PAT (purity: 99%; concentration: 100.4 ± 1.4 µg/mL), OTA (purity: 99.5%; concentration: 10.02 ± 0.08 µg/mL), and AOH (purity: 98.5%; concentration: 100.0 ± 2.2 µg/mL) used for the analysis as well as certified isotope-labelled standards U-[^13^C_7_]-PAT (purity: 98.4%; concentration: 25.79 ± 0.7 µg/mL), U-[^13^C_20_]-OTA (purity: 99.2%; concentration: 10.0 ± 0.2 µg/mL), and U-[^13^C_18_]-zeralenone (purity: 98.8%; concentration: 25.1 ± 0.4 µg/mL) were all solved in acetonitrile and were purchased from Romer Labs (Butzbach, Germany). The exact mass concentration of standard solutions was additionally determined by checking with UV spectroscopy (spectrophotometer Shimadzu UV-1700, Germany) and subsequent quantification with published molar absorptivity values (PAT: AOAC 1995; OTA: Cole and Cox 1981, Rasch 2010; AOH: Thomas 1961).

### Preparation of mixtures of standard solution and isotope-labelled solution

From the certified standards, a mixture of isotope-labelled OTA, PAT, and zearalenone with a final concentration of 0.5 µg/mL per mycotoxin was prepared in acetonitrile. Likewise, a calibrant mixture of unlabeled OTA, PAT, and AOH with a concentration of 1.0 µg/mL per mycotoxin in acetonitrile was prepared from the certified stock solutions.

### Preparation of calibration solutions

The matrix-matched calibration standards were prepared by diluting 1 mL of a cloudy apple juice with a factor of 8 by adding 7 mL of the starting mobile phase which is prepared by adding 5 mL methanol to 95 mL of water. The dilution solution contained 0.1% (v/v) acetic acid and 5 mM of ammonium acetate. The diluted juice was vortexed for 1 min with neoVortex® mixer (neoLab Migge GmbH, Heidelberg, Germany) and then left for 20 min for homogenization and afterwards centrifuged at 3005 relative centrifugal force (RCF) (*g*) at 15 °C (Megafuge 16, Heraeus, Thermo Fisher Scientific, Waltham, USA). An aliquot of 1 mL of the supernatant was transferred to an Eppendorf tube and mixed with appropriate volumes of the calibrant mixture to achieve concentrations of 1 to 50 ng/mL for PAT, AOH, and OTA. Finally, 50 µL of the isotope-labelled standard mixture was added. The mixture was then filtered with a 2-µm polytetrafluorethylene membrane (PTFE) filter (VWR International Co, Radnor, USA) and transferred into vials. The concentration range covered by the calibration standards was from 1 to 50 µg/L. All solutions were stored at − 20 °C in the dark and could be used for at least 2 months. Stability was routinely checked by measuring independent standard solutions in each sequence of LC–MS/MS measurement.

### Preparation of standard solutions for spiking experiments

Stock solutions of OTA (680 µg/mL) and PAT (780 µg/mL) were prepared in acetonitrile, whereas AOH (890 µg/mL) was prepared in acetonitrile/methanol (50/50, v/v). All solutions were stored at − 20 °C in the dark until further use.

### Sample preparation

Samples of the juice were prepared as triplicates as follows. An aliquot of 0.5 mL juice was diluted with a factor of 8, by adding 3.5 mL of the starting mobile phase of the LC system. The sample then was vortexed for 1 min with neoVortex® mixer and then left for 20 min for homogenization. The diluted juice was then centrifuged at 3005 RCF (*g*) at 15 °C using the same equipment as described for the calibration solutions. From the supernatant, 1 mL was transferred to an Eppendorf tube and mixed with 50 µL (0.5 µg/mL) of the isotope-labelled standard mixture and then filtered with a 2-µm PTFE filter. The samples were analysed by LC–MS/MS as described below.

### LC–MS/MS analyses

LC–MS/MS was performed on a Shimadzu Nexera system (Shimadzu, Kyoto, Japan) coupled to a Q-Trap 6500 + system (AB Sciex, Foster City, CA, USA) equipped with an IonDrive™ Turbo V electrospray ionization (ESI) source. Analytes were separated on a polar guard coated RP-C18 column (100 mm × 2 mm, i.d., 5 µm Gemini NX C18, with a C18-guard column) purchased from Phenomenex (Aschaffenburg, Germany). The column was kept at 40 °C. The binary gradient system consisted of (A) water and (B) methanol both being mixed with 0.1% (v/v) acetic acid and 5 mmol/L ammonium acetate at a flow rate of 0.3 mL/min. The gradient was started and held at 5% B for 1 min, was raised linearly from 5% B to 95% B during the next 8 min, and then maintained at 95% B for 5 min. Next, the mobile phase returned to 5% B within 0.5 min and the system was equilibrated for 2.5 min before the next run. The injection volume was 10 μL. Mass spectrometric detection was performed in positive and negative ESI mode in one run, and multiple reaction monitoring (MRM) was applied as scan type. The ion source parameters for the negative mode were set as follows: curtain gas 40 psi, CAD (collision activated decomposition) gas pressure medium, ion spray voltage − 4500 eV, spray gas 50 psi, dry gas 65 psi, and temperature 300 °C. The ion source parameters for the positive mode were set as follows: curtain gas 20 psi, CAD gas pressure high, ion spray voltage 4500 eV, spray gas 60 psi, dry gas 35 psi, and temperature 300 °C. MS parameters were optimized by direct infusion of each standard solution (50 ng/mL to 1 μg/mL) into the source. Each juice sample was analysed by LC–MS/MS as duplicate, i.e. was injected twice. Analyst QS Software version 1.6.3 and Multiquant version 3.0.2 (Sciex, Foster City, CA, USA) were used for analyses and quantification of the data obtained. The ESI–MS/MS parameters used are shown in Table 1. For quantification, the calibration curve was drawn by plotting the quantifier peak area ratio of each mycotoxin and that of the corresponding isotope-labelled standard (= *y*-axis) against concentration ratio of each mycotoxin and its isotope-labelled standard (= *x*-axis). The slope and the intercept were calculated by linear regression.

**Table 1:** Electrospray ionization tandem mass spectrometry (ESI–MS/MS) parameters; selected ion transitions with optimized collision energies (CE), collision cell exit potential (CXP), and declustering potential (DP) for each analyte

Analyte	Q1 mass (*m/z*)	Q3 mass (*m/z*)	DP (V)	CE (V)	CXP (V)	Retention time (min)
AOH^*^	257.0	213.0^1^	− 65	− 30	− 11	7.24
	257.0	147.0	− 65	− 42	− 11	7.24
PAT (AcOH) adduct^*^	213.0	153.0^1^	− 30	− 8	− 11	2.86
	213.0	109.0	− 30	− 16	− 5	2.86
PAT IL^*^	220.1	160.0	− 30	− 10	− 7	2.84
Zearalenone IL^*^	335.2	185.1	− 75	− 40	− 9	7.87
OTA^†^	404.0	239.0	130	10	46	7.46
	404.0	358.0^1^	130	10	15	7.46
OTA IL^†^	424.2	250.1	130	10	46	7.46

*AcOH* acetic acid, *IL* isotope-labelled

^*^ESI negative mode

^†^ESI positive mode

^1^Quantifier

### Method validation

As part of the method validation, the parameters selectivity, linearity, repeatability (relative standard deviation (RSD) calculated from results generated under repeatability conditions, RSD intra-day), intermediate reproducibility (relative standard deviation calculated from results generated under within-laboratory reproducibility conditions, RSD inter-day), recovery, and limit of quantification (LOQ) were determined for the three mycotoxins of interest (Table 2). The selectivity was assessed by comparing MRM chromatograms of blank and spiked samples for cloudy and clear apple juice. The linear regression model was tested after Mandel’s fitting test. Recovery was determined using spiked apple juices and was calculated by using matrix-matched calibration (*R*_MMC_). In addition, spiked apple juice was run as a quality control (QC) sample in the measurement series. The LOQ was determined according to the “Guidance document on the estimation of LOD and LOQ for measurements in the field of contaminants in feed and food” (Wenzl et al. 2016).

**Table 2:** Repeatability (relative standard deviation RSD intra-day, *n* = 10) and intermediate reproducibility (RSD inter-day, *n* = 3, two operators), recovery obtained from spiked apple juice by matrix-matched calibration (*R*_MMC_) each in percent and limit of quantification (LOQ) in microgram per litre

Sample	Analyte (concentration)	RSD intra-day (%)	RSD inter-day (%)	*R*_MMC_ (%)	LOQ (µg/L)
Clear apple juice	AOH (200 µg/L)	11.1	11.7	102	4.6
	PAT (200 µg/L)	9.5	10.9	93	13.3
	OTA (30 µg/L)	9.7	9.3	83	1.4
Cloudy apple juice	AOH (200 µg/L)	2.8	4.1	96	1.8
	PAT (200 µg/L)	3.3	3.4	89	7.5
	OTA (30 µg/L)	5.5	6.9	88	1.3

### Laboratory scale apple juice processing experiments

The laboratory-scale experiments were performed at Geisenheim University. After manually sorting to include only fully ripe, uninjured fruits, a total of ca. 5 kg of apples was mashed using a grinding mill (AMOS, Leingarten, Germany) and dejuiced with a tincture press (HAFICO, Fischer Maschinenfabrik, Neuss, Germany). Three samples (50 g each) of the freshly pressed juice were sampled and stored at − 20 °C in 250 mL vessels (Nalgene products HDPE, Thermo Fisher Scientific Inc, New York, USA) until further analysis. Subsequently, aliquots of the standard solutions of PAT, AOH, and OTA were added to 1 L of the freshly pressed juice. The concentrations of the mycotoxins before and after spiking are shown in Table 3. Samples of 50 g were taken after mycotoxin addition and stored at − 20 °C in 250 mL vessels, as described before, to determine the actual concentration. Subsequently, the mycotoxin-enriched juice was depectinized with a pectinolytic enzyme preparation (Fructozym P) according to the specification of the manufacturer (7 mL/hL, ambient temperature, 2 h), and sampled again for mycotoxin analysis as described above. Afterwards, the juice was fined by consecutively adding gelatin (12 g/hL), colloidal silica (60 mL/hL), and bentonite (50 g/hL), followed by centrifugation (2800 × *g*, model 5804, Eppendorf, Hamburg, Germany). Subsequently, the centrifugated juice was sampled again for the mycotoxin analysis. These experiments were performed in technical triplicate (*n* = 3).

**Table 3:** Mycotoxin concentration in unspiked juice and the measured concentration (each in microgram per litre) in the spiked juice for the laboratory-scale and the pilot-plant-scale experiment

Analyte	Laboratory-scale experiment		Pilot-plant-scale experiment	
	Unspiked juice (µg/L)	Spiked juice (µg/L)	Unspiked juice (µg/L)	Spiked juice (µg/L)
PAT	< LOQ	104	263	399
OTA	< LOQ	82	< LOQ	64
AOH	< LOQ	127	< LOQ	90

*LOQ* limit of quantification

### Pilot-plant-scale apple juice processing experiments

Pilot-plant-scale apple juice processing was performed at Geisenheim University. For this purpose, a total of ca. 200 kg of apples were mashed directly, without previous sorting or washing steps, using an eccentric screw pump with an integrated stationary cutting mechanism (open hopper pump BTM, Seepex, Bottrop, Germany). Afterwards, the mash was pressed with an HP-L 200 hydraulic horizontal filter press (Bucher, Niederweningen, Switzerland) yielding ca. 130 L juice of which 100 L (105 kg) were spiked with PAT, AOH, and OTA. Mycotoxin levels before and after spiking are shown in Table 3. Prior to sampling, the spiked juice was rigorously stirred manually with an impeller paddle. The freshly pressed, “spiked” juice was then processed in 10–50 L batches per processing line as illustrated in Fig. 1 and according to commercial practice as described below.

For the pilot-plant scale production of cloudy apple juice (line 1), an aliquot of 20 L of the spiked batch was centrifuged (SA1-02–175, GEA Westfalia, Oelde, Germany) and flash-pasteurized by heating the juice to 72 °C within ca. 0.5 min using a fruit juice dispenser (PAS1-PS2-81-V2, MABO, Eppingen, Germany) and subsequent hot-filling into the aforementioned 250 mL vessels prior to cooling to room temperature.

For the production of clear apple juices (lines 2–6), the remaining 80 L juice was treated with a pectinolytic enzyme preparation (Fructozym P, 7 mL/hL, ambient temperature, 2 h), divided into different batches and subjected to various clarification procedures as indicated in Fig. 1. Aliquots of 10 L of the depectinized juice were fined with 50 g bentonite and 30 g activated charcoal per hL of juice (line 2), with 30 g pea protein and 150 g colloidal silica per hL (line 3), and with 20 g potato protein per hL (line 4), each conducted according to specification of the manufacturers of the fining agents. Fining of lines 2–4 was followed by cross-flow ultrafiltration (Romicon PM 100, Koch Membrane Systems, Aachen, Deutschland) with a cut-off at 100 kDa. The yielded juices were subsequently pasteurized as described for line 1. A further, larger aliquot of 50 L juice was fined with the most common fining agent combination, i.e. with 12 g gelatin, 60 mL colloidal silica, and 50 g bentonite per hL of juice. Subbatches of the latter juice were then filtered either using ultrafiltration (line 5, 10 L juice) or sheet filtration (line 6, 40 L juice, 20S Filter Cartridge, Seitz (Pall), Bad Kreuznach, Germany) through three filter sheets (Becopad 270, Eaton) prior to pasteurization like described for line 1.

Processing was done without technological repetition (*n* = 1). Three samples (each 50 g) were taken along the different processing lines at several stages of the processing using 250 mL vessels (Nalgene products HDPE, Thermo Fisher Scientific Inc, New York, USA) and kept at − 20 °C until analysis (Fig. 1).

### Data analysis

**Fig. 2:**
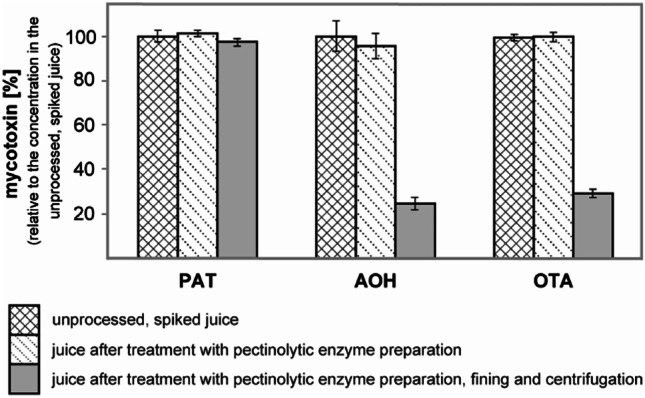
Effect of enzymatic treatment (pectinolytic enzyme preparation) and fining (bentonite, gelatine, and colloidal silica) followed by centrifugation on mycotoxins as observed in preliminary laboratory-scale experiments in threefold technical repetition. The initial concentrations of mycotoxins after spiking were 104 µg/L patulin (PAT), 127 µg/L alternariol (AOH), and 82 µg/L ochratoxin A (OTA)

Results were expressed as means ± standard deviation unless stated otherwise. In laboratory-scale experiments, the means represent results from three fully independent technical replicates and standard deviations refer to the variability of results between these replicates (cf. Fig. 2).

**Fig. 3:**
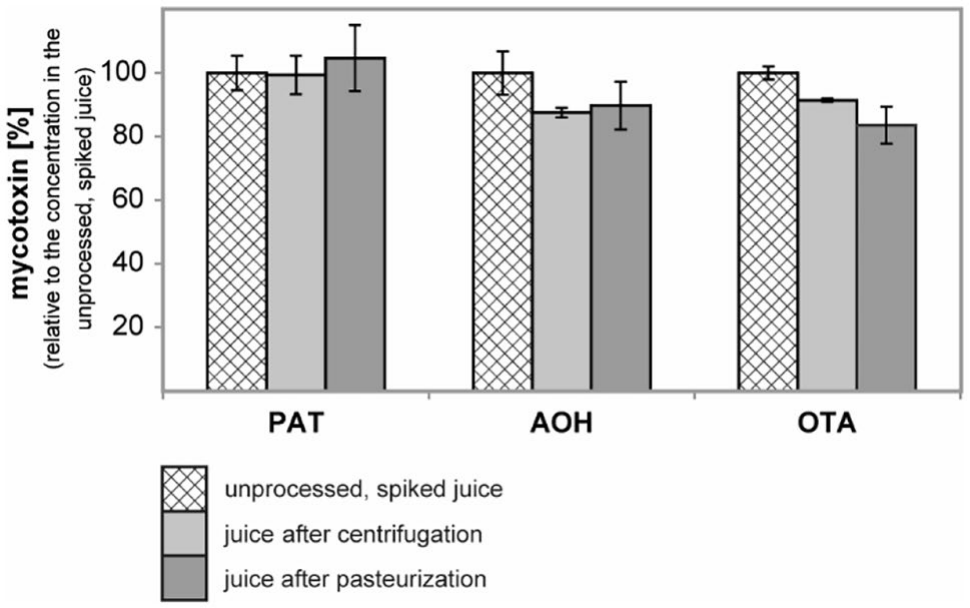
Effect of cloudy apple juice processing on mycotoxins (processing steps correspond to line 1 in Fig. 1) as observed in pilot-plant scale experiment without independent technical repetition. Error bars represent standard deviations based on the analysis of multiple samples (*n* = 3) at the specific sampling points. The initial concentrations of mycotoxins after spiking were 399 µg/L patulin (PAT), 90 µg/L alternariol (AOH), and 64 µg/L ochratoxin A (OTA)

**Fig. 4:**
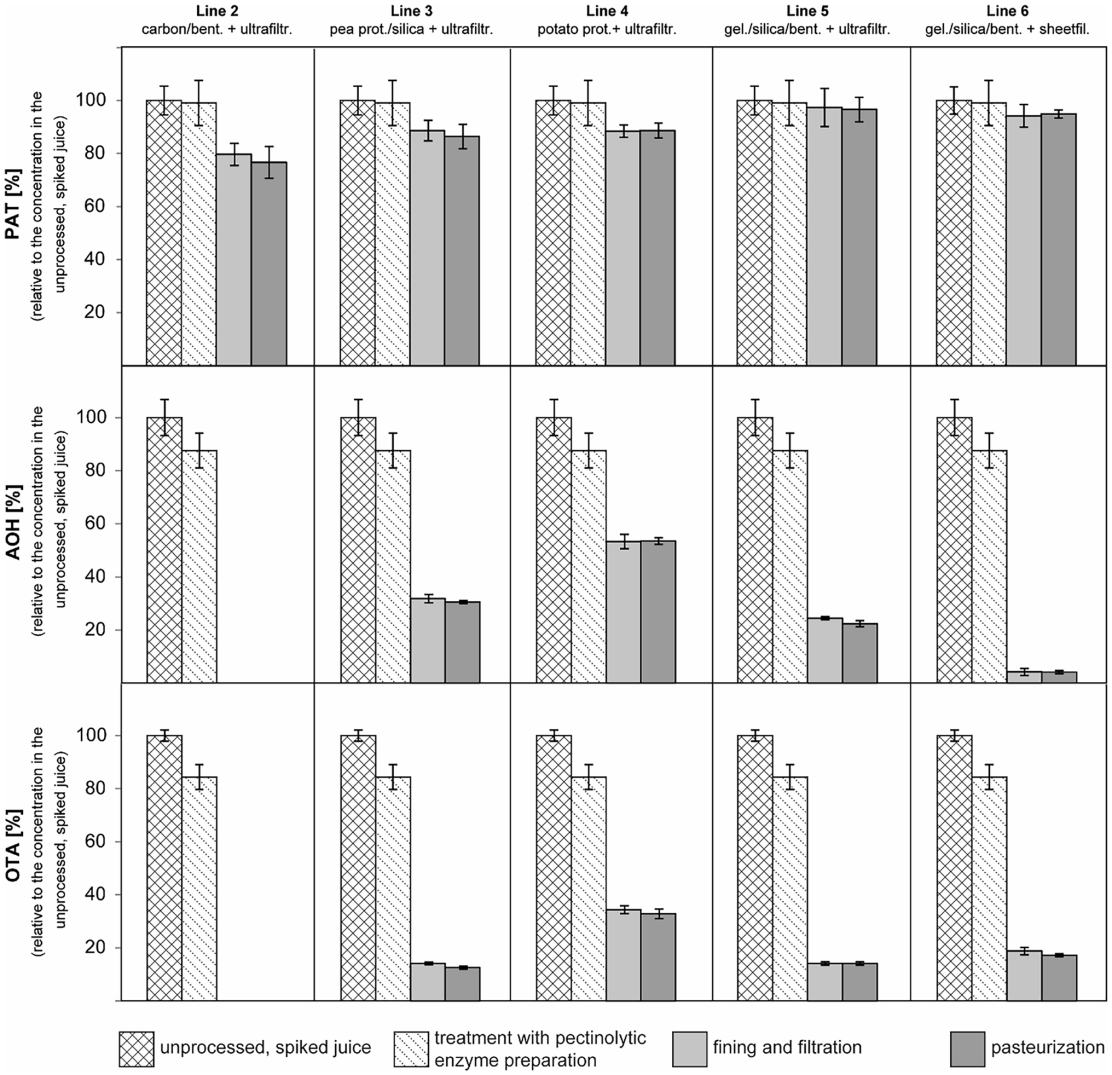
Effect of clear apple juice processing on mycotoxins (processing steps correspond to the lines 2–6 in Fig. 1). These pilot-plant scale experiments were performed without technical repetition. Error bars represent standard deviations based on the analysis of multiple samples (*n* = 3) at the specific sampling points. The following abbreviations were used for the processing aids and processing steps: carbon = activated charcoal, bent. = bentonite, pea prot. = pea protein, potato prot. = potato protein, gel. = gelatine, silica = colloidal silica, ultrafiltr. = ultrafiltration, sheetfil. = sheet filtration. The initial concentrations of mycotoxins after spiking were 399 µg/L patulin (PAT), 90 µg/L alternariol (AOH), and 64 µg/L ochratoxin A (OTA)

In pilot-plant-scale experiments, a total of three samples at each sampling point was obtained during processing of a single large 200 kg batch of apples. Means and standard deviations refer to results obtained from these three samples, which were not fully independent due to their provenance from one batch of apples (Figs. 3 and 4).

### Chemical analysis of the juice

The total soluble solids (in °Brix) were analysed by digital refractometry (Abbemat, Dr. Kernchen, Seelze, Germany) and the density by densitometry (DMA 48, Paar, Ostfildern, Germany). The pH and the total titratable acidity (calculated as citric acid at pH 8.1) were measured potentiometrically (Titroline alpha, Schott, Mainz, Germany). The total phenols were assayed with the Folin-Ciocalteu-method based on a ( +)-catechin calibration using a Konelab 20 Xtr analyser (ThermoFisher, Dreieich, Germany).

## Results and discussion

### Analytical method performance

The evaluation of the performance of the method was determined for the three mycotoxins in cloudy and clear apple juice by performing an inhouse-validation study. Results of the analyses were compliant with the analytical performance criteria of the European Committee for Standardization (CEN) for single laboratory validated methods of analysis for the determination of mycotoxins (CEN 2010) (Table 2). All recoveries of the QC samples were in good agreement with the analytical performance criteria of Commission Regulation (EC) No 401/2006 (EC 2006b).

**Fig. 5:**
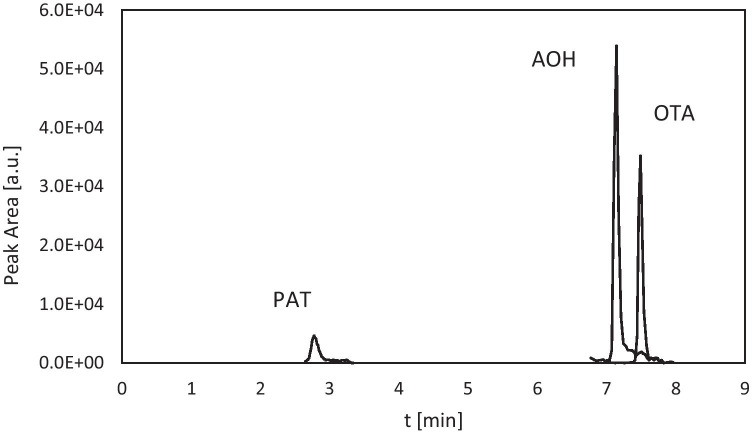
Extracted ion chromatograms of mycotoxins: matrix-matched calibration standards of patulin (PAT), ochratoxin A (OTA), alternariol (AOH) at 5 µg/L each

Selectivity: There were no interfering co-eluting peaks detected for any of the target analytes in both clear and cloudy juices. Examples of extracted ion chromatograms for matrix-matched calibration standards of PAT, AOH and OTA are shown in Fig. 5.

Linearity: Regression coefficients were between 0.98 and 0.99 in a range of 1.6–100.0 µg/L in the matrix-matched calibration curves for PAT and AOH and in the range of 0.3–18.0 µg/L for the OTA.

Precision: The RSD intra-day and RSD inter-day ranged between 2.8–11.1% and 4.1–11.7%, respectively.

Recovery: The recoveries were between 83 and 102%.

Limit of quantification: The LOQ varied between 1.3 and 13.3 µg/L.

### Laboratory-scale apple juice processing experiments

The preliminary laboratory-scale experiments focused on the effect of the treatment with a pectinolytic enzyme preparation and the most common fining combination (gelatine, colloidal silica, and bentonite) on the three mycotoxins PAT, AOH, and OTA. The freshly pressed apple juice was characterized by a pH of 3.4, total titratable acidity of 6.1 g/L, total phenol content of 174 mg/L in average, a density of 1.0485 g/cm^3^, and total soluble solids of 12.2°Brix. Mycotoxin levels in the fresh, raw apple juice were all below our LOQ prior to mycotoxin addition and then were 82 µg/L for OTA, 127 µg/L for AOH, and 104 µg/L for PAT after mycotoxin addition (Table 3).

Figure 2 shows the results of the investigated processing steps. The treatment with a pectinolytic enzyme preparation had no effect on any of the studied mycotoxins. The fining with gelatine, colloidal silica, and bentonite combined with subsequent centrifugation led to a loss of AOH by 76% (from 127 to 31 µg/L) and in the case of OTA by 71% (from 82 to 24 µg/L). Unlike concentrations of OTA and AOH, that of PAT remained unaffected by these fining agents (Fig. 2).

### Pilot-plant-scale apple juice processing experiments

Similar to the results of the preliminary laboratory-scale experiments, the pilot-plant-scale experiment confirmed that the mycotoxins react very differently toward the fining agents and processing steps (Fig. 1).

Noteworthy, neither OTA nor AOH were found in the unspiked juice produced at pilot-plant scale (pH of 3.5, total titratable acidity of 4.0 g/L, total phenol content of 341 mg/L, density of 1.0520 g/cm^3^, and total soluble solids of 12.9°Brix). Both OTA and AOH were below our LOQ in the raw apple juice before adding the mycotoxins. After mycotoxin addition, the concentration of OTA in the spiked apple juice was measured at 64 µg/L and that of AOH at 90 µg/L (Table 3). Although a certain genuine PAT concentration had been expected due to the intended omission of washing and manual sorting of the fresh apples prior to processing, an unexpectedly high level of 263 µg/L was determined in the unspiked juice, which is more than five times the maximum level of PAT in apple juice according to EU regulations (EC 2006a). After spiking, the concentration of PAT in the apple juice was 399 µg/L (Table 3).

Cloudy apple juice processing (line 1) and its influence on PAT, OTA and AOH (Fig. 3): Centrifugation and pasteurization had no influence on the PAT concentration. The two other mycotoxins, AOH and OTA, showed only a slight reduction after centrifugation. No effect was observed by pasteurization for AOH, but a further slight reduction in case of OTA. In total, the processing of cloudy apple juice led to a loss of AOH by 10% (from 90 µg/L to 81 µg/L) and a loss of OTA by 16% (from 64 µg/L to 54 µg/L). No reduction was observed for PAT.

Clear apple juice production by different processing lines (lines 2–6) and its influence on OTA (Fig. 4): The OTA concentration was only slightly reduced after enzymatic treatment (pectinolytic enzyme preparation) from 64 to 54 µg/L (reduced to 84%). The processing line 2, using activated charcoal in combination with bentonite and ultrafiltration, was most effective in reducing the initial level of OTA. The classical fining agents gelatine, colloidal silica, and bentonite in combination with ultrafiltration were effective, as well. Pasteurization had no influence on the OTA amount at all. In total, processing lines 3, 4, 5, and 6 led to a loss by ca. 88%, 67%, 86%, and 83%, respectively (the initial concentration of 64 µg/L was reduced to 8, 21, 9, and 11 µg/L). No detectable amounts of OTA were found in the juice processed by processing line 2.

Clear apple juice production by different processing lines (lines 2–6) and its influence on AOH (Fig. 4): Treatment with a pectinolytic enzyme preparation reduced AOH only slightly from 90 to 79 µg/L. Comparable to OTA, the AOH levels were reduced drastically to undetectable levels during clear apple processing when activated charcoal in combination with bentonite followed by ultrafiltration were applied (processing line 2). Similarly, treatment with gelatine/colloidal silica/bentonite in combination with ultrafiltration and sheet filtration (processing lines 5 and 6) reduced the AOH content.

The use of plant proteins was the least effective method for the reduction of AOH (processing line 3 and line 4), though the following ultrafiltration in combination with pea protein and colloidal silica was more effective than potato protein and ultrafiltration. The subsequent pasteurization had no effect on the AOH concentration in none of the processing lines. Following total reduction was found for AOH (initial concentration: 90 µg/L) by the use of processing lines 2–6: < LOQ, 70% (to 27 µg/L), 47% (48 µg/L), 78% (20 µg/L), and 96% (4 µg/L), respectively.

Clear apple juice production by different processing lines (lines 2–6) and its influence on PAT (Fig. 4): The enzymatic treatment had no effect on PAT (initial concentration: 399 µg/L; after enzymatic treatment: 396 µg/L). The PAT level was reduced by 20% (from 396 to 318 µg/L) after treatment with activated charcoal/bentonite combined with ultrafiltration (processing line 2). These fining agents were the most effective ones in reducing PAT. No effects were observed for the processing lines using gelatine/colloidal silica/bentonite in combination with ultrafiltration or sheet filtration (processing lines 5 and 6). Only a slight tendency for a reduction was observed for the processing lines using enzymatic treatment, plant proteins, ultrafiltration and pasteurization (14% using processing line 3 and 11% using line 4: from 399 to 345 and 354 µg/L, respectively).

### Comparison of the results of both processing studies

No mycotoxins were found in the unspiked juice, used for the preliminary laboratory-scale processing experiment, and PAT was the only naturally occurring mycotoxin found in the juice, processed for the pilot-plant-scale experiments. The already high PAT concentration in the unprocessed juice of 263 µg/L might have been caused by the fact that the apples as used in this experiment for processing were not further sorted, washed, and trimmed to remove mouldy segments prior to pressing. These findings highlight the great importance of sorting, washing, and trimming for producing safe apple juices. In a previous study, Sydenham et al. (1995) showed that high amounts of naturally occurring PAT in the fruit can be minimized by washing and trimming, resulting in a reduction of PAT of up to 86%. The pilot-plant-scale experiment was performed in April using apples that had been cold-stored for almost 6 months after harvest in October. Stored apples bear a higher risk of having fungal infected cores even though such apples have been shown to be often in apparently good order and condition (Soliman et al. 2015). Further, the PAT production of some *Penicillium expansum* strains was reported to be even stimulated at cold conditions (Baert et al. 2007). Hence, the utilization of cold-stored apples as well as the omission of sorting, washing, and trimming might have resulted in the above-mentioned, increased levels of PAT.

The presented data clearly showed that cloudy apple juice processing steps had negligible effects on the mycotoxin concentration, whereas clarification led to a reduction, depending on the mycotoxin and the processing step used for clarification. The production of cloudy apple juice includes two major steps after de-juicing: centrifugation and pasteurization. This study showed that the effect of centrifugation was negligible for the three investigated mycotoxins. Only OTA and AOH showed a slight decrease of 8–12% (from 64 to 59 µg/L and from 90 to 79 µg/L, respectively). Bissessur et al. (2001) have earlier studied the influence of various processing steps like pressing, centrifugation, and fining with bentonite only on the retention of PAT, which had been spiked into an apple mash at 2000 µg/L. They found the most pronounced reduction of PAT by 52.5% upon pressing. Subsequent centrifugation reduced PAT levels by 20.5%, being explained mainly by the removal of the particles to which PAT had been bound (Bissessur et al. 2001). Since Bissessur used a five-fold higher PAT dose, a direct comparison of these results with our findings is intricate.

Furthermore, the effect of pasteurization on the mycotoxin levels of the cloudy apple juice was negligible. Likewise, all mycotoxins were thermally stable against pasteurization in all performed experiments in case of clear apple juice processing (*n* = 5, line 2–6). This result is comparable to an earlier study, which found that OTA is stable up to 180 °C depending on the matrix components (Raters and Matissek 2008). The thermal stability of PAT was investigated in further studies, but with contradictory results (e.g. Leggott et al. 2000; Kabak 2009). Heatley and Philpot (1947) reported PAT to be stable at 100 °C/15 min at a pH-value of 2. In contrast, other authors reported an inverse correlation between the PAT content in the juice and the temperature used for concentration (Kadakal and Nas 2003). Further, the pH has been shown to influence the stability of PAT; i.e. higher pH may lead to accelerated degradation of PAT (Lovett and Peeler 1973). Other studies considered PAT to be thermostable in fruit juices with a low thiol content (Scott and Somers 1968). While AOH was found to be significantly unstable at high temperatures such as 100 °C and 110 °C during the processing of tomato products (Estiarte et al. 2018), it remained stable during the pasteurization of the cloudy and clear apple juice at 72 °C as performed herein. In brief, the processing steps applied during processing of the freshly pressed, raw juice into a cloudy apple juice (i.e. centrifugation, pasteurization) did not allow effectively reducing OTA, AOH, and PAT concentrations. Thus, mycotoxin concentrations found in cloudy apple juices might allow direct conclusions about the used raw materials and if careful sorting, thorough washing and effective trimming had been conducted.

Production of clear apple juices requires a series of further process steps not being used for cloudy apple juice manufacture, i.e. the so-called clarification commonly consisting of three technological steps: an enzymatic treatment, an adsorptive removal of undesired compounds with certain fining agents, and a filtration step. First, the treatment with the pectinolytic enzyme preparation for 2 h at ambient temperature (ca. 20 °C) had almost no effect on the mycotoxin levels in the preliminary laboratory-scale experiment (*n* = 3), as well as in the pilot-plant-scale experiment (*n* = 1). Only OTA and AOH were slightly reduced in the pilot-plant-scale experiment (Fig. 4), while in the preliminary laboratory-scale experiment, no or only a small effect was observed (Fig. 2). A previous study has shown that several commercial proteolytic enzyme preparations had allowed hydrolytic cleavage of OTA’s amide bond, i.e. of OTA into ochratoxin α (Abrunhosa et al. 2006). In our study, the treatment with a pectinolytic enzyme preparation seemed to expectedly have only a negligible effect on OTA. While PAT levels also remained unchanged upon treatment with a pectinolytic enzyme preparation in our lab- and pilot-plant-scale experiments, Bissessure et al. (2001) have reported that a treatment with a pectinase from *A. niger* for 2 h at 40–45 °C apparently had slightly reduced the PAT content of apple juice by 4.5%. However, the remaining PAT content recovered after enzymatic treatment was still very high (about 550 µg/L; Bissessure et al. 2001). Studying samples of sixteen lots from an industrial apple juice production line, Welke and co-workers (2009) have reported that the samples obtained after the enzymatic pectinase treatment step had PAT contents (mean: 188 µg/L; range: 91–244 µg/L) being 28% lower than those of the preceding processing step (mean: 262 µg/L; range: 131–406 µg/L). The report does not provide details about the enzymatic treatment such as specification of the used enzyme preparation, the duration of the treatment, and the applied temperatures. In addition, technical details about the apple juice production process and the sampling procedure remain too unclear (Welke et al. 2009) for a direct comparison with the data obtained from our study, showing that the treatment with a pectinolytic enzyme preparation had virtually no influence on the PAT levels during apple juice production.

Among the tested fining agents and filtration methods, activated charcoal/bentonite combined with ultrafiltration was the most effective method in reducing the amount of the studied mycotoxins. AOH and OTA were removed completely, whereas PAT was reduced less efficiently (approx. 20%). This result may have been influenced by the high level of naturally occurring PAT. In a previous study, Kadakal and Nas (2002) found that PAT can be reduced significantly (by 57%; from 62.3 to 26.7 µg/kg) by stirring apple juice with 3 g activated charcoal per litre juice for 30 min followed by a filtration through filter paper to remove the activated charcoal. The same study suggested that the duration of treating apple juice with activated charcoal did not affect the efficiency (Kadakal and Nas 2002). Furthermore, the dosage applied in the mentioned study was tenfold higher than in our study. Since activated charcoal was used in combination with bentonite, the influence of the individual agents cannot be considered separately. However, bentonite has been described to reduce mycotoxins, as well. For instance, bentonite is often used as a fining agent in the clarification of white wine. Anli et al. (2011) reported that bentonite led to a decrease of the OTA by 20%. The same study showed that the combination of bentonite with gelatine or gelatine/casein did not further reduce OTA values. On the other hand, the presented data here confirm the efficient removal of OTA, if bentonite in combination with gelatine and colloidal silica is applied. This processing step reduced OTA even by 71% (from 82 to 24 µg/L) in the laboratory-scale trial (*n* = 3) and by 80% (from 54 to 11 µg/L) in the pilot-plant-scale experiment (*n* = 1). Furthermore, this processing step was efficient in minimizing AOH, which was reduced by 75% (from 122 to 31 µg/L) in the laboratory-scale experiment and by 73% (from 79 to 21 µg/L) in the pilot-plant-scale trial. The combination of bentonite/gelatine/colloidal silica showed no effect on the concentration of PAT.

Fining agents based on plant proteins, which have recently been established in the fruit juice industry, were also investigated for their mycotoxin-reducing activity. In combination with ultrafiltration, AOH and OTA were reduced by the use of pea and potato protein. However, pea protein and colloidal silica combined with ultrafiltration was more efficient than potato protein. For instance, the overall reduction using this processing step in case of AOH was as follows: from 79 to 29 µg/L (reduced by 63%) for pea protein and from 79 to 48 µg/L (reduced by 39%) for potato protein. In case of PAT, only negligible effects with a reduction of approx. 11% (for pea protein from 396 to 354 µg/L and for potato protein from 396 to 353 µg/L) were observed.

In the case of the fining agent bentonite in combination with colloidal silica and gelatine, both ultrafiltration and sheet filtration were used. Results, obtained for PAT and OTA by sheet filtration were similar to those achieved by ultrafiltration. However, AOH was decreased to a higher extent by sheet-filtration than by ultrafiltration. Since this experiment was done without repetition, further experiments are required to verify these results, but a possible adsorbing activity of the cellulose based filter-sheets cannot be excluded.

In brief summary, certain processing steps in apple juice production, including centrifugation, treatment with a pectinolytic enzyme preparation. and pasteurization, were shown to have no or only negligible effects on the levels of PAT, OTA, and AOH. However, fining agents in combination with filtration were demonstrated to reduce the content of these mycotoxins in the final product more effectively.

Among the tested fining agents and filtration methods, activated charcoal/bentonite combined with ultrafiltration (processing line 2, Fig. 1) was the most effective method in reducing the amount of the studied mycotoxins. This processing step led to a reduction of AOH and OTA below the LOQ, while PAT was only reduced by 20% (from 396 to 318 µg/L). However, this was the most effective PAT-reducing processing line, observed in our experiments. Other tested processing lines had only minor or negligible effects on PAT levels.

In addition to the processing steps studied herein, several studies have suggested that new methods such as the use of gamma-irradiation, UV irradiation, or inactive yeasts have been able to eliminate or reduce PAT from apple juices (Zegota et al. 1988; Yue et al. 2011; Assatarakul et al. 2012). Biotechnological approaches, e.g. the use of antifungal biomolecules, biomolecule-based adsorbents, or enzymes, that are active in PAT degradation, are future possibilities for minimizing PAT in apples or apple-based products (Ngolong Ngea et al. 2020). Chemical adsorbents, e.g. using cholestyramine or esterified glucomannans, could be further alternatives, able to minimize the other mycotoxins, as well (Azam et al. 2021).

It should be noted that mycotoxin removal by chemical measures should not be an aim of manufacturers, being prohibited in the European Union (EC 2006a).

Manufacturers should rather focus on optimizing pre-harvest, harvest, and post-harvest conditions, as they play the key role in affecting the amount of mycotoxins in the final product. For instance, the EC as well as Codex Alimentarius (CAC) has recommended both the Good Agricultural Practice (GAP) and Good Manufacturing Practices (GMP) to reduce PAT (EC 2003; CAC 2003). These recommendations include measures promoting the production of healthy fruits and measures during the harvest and post-harvest stages to avoid the damage of the fruits and the prevention of the fungal growth and contamination (EC 2003; CAC 2003). The main strategy of maintaining a tolerable level of mycotoxins in apple juice should focus on preventing the occurrence of these mycotoxins in the raw material and, thereby, also in the final product.

While mycotoxin analyses of cloudy apple juices seem to allow direct conclusions about the used raw materials, analyses of clear apple juices are more difficult to evaluate for food control authorities.

## Data Availability

Not applicable.
